# Curriculum effectiveness for secondary-aged students with severe intellectual disabilities or profound and multiple learning difficulties in Australia: Teacher perspectives

**DOI:** 10.1177/17446295241228729

**Published:** 2024-01-24

**Authors:** Tess Rendoth, Jill Duncan, Judith Foggett, Kim Colyvas

**Affiliations:** School of Education, 5982 University of Newcastle, Callaghan, NSW, Australia; College of Engineering, Science and Environment, 5982 University of Newcastle, Callaghan, NSW, Australia

**Keywords:** curriculum, inclusion, individual education planning, intellectual disabilities, severe or profound and multiple learning difficulties, teachers

## Abstract

The active inclusion of students within education systems relies on a curriculum that caters to all. This article presents partial findings from Australian mixed methods research examining 46 teacher perspectives on the curriculum and its ability to support their practice in supporting students aged 12-19 years with severe intellectual disability or profound and multiple learning difficulties who attend specialist school settings. Results reveal that Australian teachers see the current curriculum as insufficient in its design and content and unable to cater to their students educational and social capacities or needs. Strengths essential to the reform process are highlighted, emergent challenges discussed and recommendations for future action are presented.

Since the early 1980s, inclusive education policies for students with disabilities have influenced the education of students with severe intellectual disabilities or profound and multiple learning difficulties (SPMLD) (Male, 2015). Educational inclusion policies set directives that all aspects of schooling and teaching practice can be modified, including the physical environment, curriculum, teaching methods, assessment, and reporting practices (Pearce, 2009; Strnadova et al., 2021; UNESCO, 1994). The intention of these policies is that they will lead to an equitable educational system where all children have access to the same curriculum, social and physical experiences and receive support to access them on the same basis as others (Pearce, 2009). This paper considers these goals and investigates if current teacher perspectives in relation to the current Australian curriculum feel it enables or limits these policy intents, and more broadly, the educational and social inclusion for their students.

The Australian education system prioritises inclusivity, adhering to the United Nations Salamanca Statement and supported by national legislation, The Disability Discrimination Act 1992 (DDA) and The Disability Standards for Education 2005 (DSE) (Australian Government, 2022a, 2022b). When reflecting on the success of such policies, we must consider the experiences of inclusion felt by those students with the most complex needs and their teachers. Students with severe intellectual disabilities or profound and multiple learning difficulties are likely to have a combination of the following characteristics: severe intellectual disabilities, emergent and idiosyncratic communication profiles, non-traditional sensory processing, physical disabilities, complex medical profiles and/or challenging behaviours. These students will need elevated levels of adult and peer support throughout their lives (Colley and Tilbury, 2022; Nind & Strnadova, 2020). The terminology used to describe these students internationally can often include 'students with extensive support needs' or 'complex learners'. In Australia, most students with severe intellectual disabilities or profound and multiple learning difficulties attend specialist schools, which are self-contained schools that serve the needs of students with similar disabilities exclusively.

## Introduction

Curriculum entitlement is one of Australia's critical expressions of inclusion at a policy and structural level. Curriculum entitlement is the dedication that all students have access to the same curriculum so that all students' educational experiences and growth are as similar as possible. There is a shared agreeance that curriculum entitlement can be a primary mechanism to increase social inclusion, decrease social and emotional exclusion, and ensure that all students are rightfully acknowledged as valuable contributors to the community (Colley, 2018; Pennington et al., 2016; Walker et al., 2018). As supported by governments' approach to inclusive education, all students having access to the same curriculum is an overall social justice intent of OECD nations that should be actively pursued (OECD, 2022).

With curriculum entitlement at the centre of Australia's approach to inclusive education, defining what an educational curriculum represents is essential. Pinar (2004) posits that curriculum is the symbolic and practical representation of our geopolitical and social values. Australia’s curriculum is reflective of the work of Tyler (1949), whose outcomes model (now referred to as the objectives model) emphasises that curriculum is to be linear and contain prescribed patterns of study and specific content, adopting an outcomes-based evaluation and assessment model centrally endorsed by an educational authority (Lunenberg, 2011). This curriculum style aims for stability and rigidity, attempting to maintain functional and historical continuity in responding to emergent or changing socio-political and economic trends in the future (Wallin, 2011). These structural factors are at odds with the conceptual and philosophical individualised planning required to meet the needs of students with disabilities and for students with severe intellectual disabilities or profound and multiple learning difficulties more specifically (Lawson and Jones, 2018). In adopting the Salamanca Statement, the homogeneity of our standardised system was challenged by the need to include students with disabilities, mandating general curriculum access as key to the expression of inclusive practice.

The structure of curriculum in Australia combines two levels of standardised curriculum, with The Australian Curriculum, provided by The Australian Curriculum and Reporting Authority (ACARA), and then each state authority having its own curriculum that draws from The Australian Curriculum but is locally interpreted. The Australian Curriculum also included the Early Years Learning Framework for pre-kindergarten development, and the Literacy and Numeracy Progressions to support curriculum design in these areas for students. State curriculums then include an internal ‘parallel’ curriculum or modified outcome options specifically designed to use when planning for students with disabilities. To make these curriculums further personalised to the needs of students, all authorities promote further curriculum adaptation as a directive for teaching staff.

## Individualisation: Tensions and challenges

All educational authorities in Australia advise education personnel on producing individualised education plans (IEPs) for students with disabilities. ACARA (2022) identifies curriculum modification as one of three primary avenues to personalise learning, with pedagogical and environmental modifications also acknowledged.

The role that student-led practices have within the design of IEPs and curricula for students with severe intelelctual disabilities or profound and multiple learning difficulties is where the differences between these two directives, one of educational standardisation and the other of student individualisation, are in direct tension (ACARA, 2022; Cheng-Man Lau, 2001; NSW Education Standards Authority, 2022a). Under the directive of reasonable adjustments outlined in the DSE, IEPs are student-led individualised curriculum interpretations, centralising the needs and capacities of the individual rather than the designations or priorities of the general curriculum and its pre-determined outcomes (Education Victoria, 2022). This process of curriculum adaptation recognises the agency of individual teachers and centres student needs in teacher decision-making. IEPs enable the possibility of curriculum to be an active element in promoting high expectations and responding to the current and emerging socio-political and cultural needs of the individual students (Cheng-Man Lau, 2001; Doll, 1993; Taba, 1962).

IEPs are designed to recognise student preferences, feelings, life goals and rich personal histories and to maintain equitable contribution by all parties, including the student (Matthews, 2015). IEPs should focus on skills development to support a student's independence in a student-centered decision-making framework (Victorian Department of Education and Training, 2022).

Curriculum planning for students with severe intellectual disabilities or profound and multiple learning difficulties directly expresses tension and contradiction within a standardised structure (Lawrence-Brown, 2004; Tomlinson, 1999; Walker et al., 2018). Teachers are positioned in the middle of this negotiation and are responsible for maintaining a balance between the demand of curriculum entitlement and for implementing individualised curriculums for their students. Datta (2019) and Walker et al. (2018) discuss that there is currently no curriculum advice or guidance at any state or national level relevant to supporting teachers of students with severe intellectual disabilities or profound and multiple learning difficulties in this process of individualisation.

The interaction between the curriculum and the directive set to teachers to individualise curriculum for students with severe intellectual disabilities or profound and multiple learning difficulties must be investigated to see if the current curriculum can effectively support positive educational outcomes for students and their teachers and how we can increase curriculum effectiveness (Australian Curriculum Assessment and Reporting Authority, 2012; Lacey et al., 2015; Moljord, 2018; Walker et al., 2018). The capacity of the curriculum to offer this support is of particular importance due to the enrolment of most students in specialist settings, which are the most geographically and socially isolated from mainstream contexts (Strnadova et al.; 2021).

## Research Question

The present research examines attitudinal perspectives, and the current levels of engagement teachers have with the curriculum when planning curriculums for secondary-aged students with severe intellectual disabilities or profound and multiple learning difficulties. *How are the currently endorsed curriculums and supporting documents perceived, adjusted and/or operationalised by teachers who support students aged 12-19 with severe intellectual disabilities or profound and multiple learning difficulties who attend specialist settings?*

## Methodology

### Procedure

The initial survey instrument was developed through an iterative process first by the research team and then forwarded to representatives of peak organisational bodies within the sector for consultation. These bodies included state and national level representatives from the Australian Special Education Principals Association (ASEPA), the Australian Association of Special Education (AASE) and the Special Education Principals and Leaders Association (SEPLA). As a survey of this kind has yet to be done in Australia, this process ensured the survey tool correlated to teachers’ contemporary professional and practice contexts. Participants were recruited through peak organisational bodies within the sector (as cited above) and other professional networks. Recruitment was via email distribution through peak bodies to their membership, and posting on specific social media pages by the researchers.

The survey was accessible online for a four-month period. The first section of the survey was dedicated to screening participants as per the inclusion criteria listed above. Although over 1600 people initially commenced the screening process, 51 were deemed eligible in meeting both requirements of eligibility if they a) had primary curriculum planning responsibility for at least one student aged 12-19 years with severe intellectual disabilities or profound and multiple learning difficulties and b) worked as a teacher in a government school for specific purposes/specialist educational setting, 46 completed the survey.

### Materials

The survey consisted of four sections, a) Demographics and experience, b) Personal and professional identities, c) Curriculum use and d) Professional learning and support. There were 76 questions, not all of which are reported herein. Sections a) and b) of the survey were shown to all participants. Branching logic was used throughout the remaining elements of the survey, which meant that participants only saw specific questions if they answered in certain ways to others. For example, the participant would not see questions about the Australian Curriculum if they indicated they did not use it in their practice. The survey was designed using a mixed-methods approach, with both qualitative and quantitative data collected.

The University of Newcastle's Human Ethics Research Committee H-2020-0161 approved the research.

### Analysis

**Figure 1.**
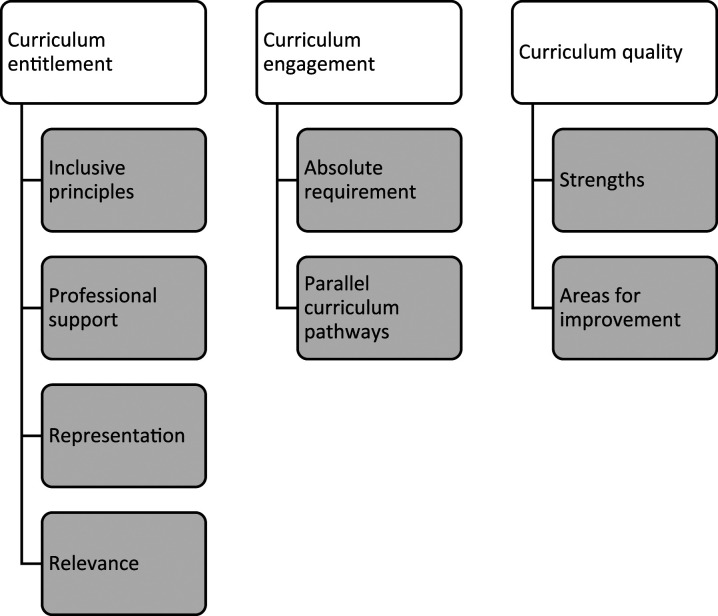
Primary Themes and Sub-themes.

Data were analysed using a mixed-methods design approach. Qualitative analysis was undertaken by the first author using the constant comparative method to consistently map thematic patterns across multiple open-ended response questions using NVivo, with authors two and three reviewing and approving the codes assigned (Glaser & Strauss, 1967; Maykut & Morehouse, 1994, QRS, 2020). Quantitative analysis was completed by descriptive analysis of all yes/no and Likert questions (5-point). The differences between curricula for each domain were examined as percentage success and tested for statistical significance using chi-square tests. Exact tests were used to ensure the reliability of significance tests due to small sample sizes and low expected values for some table combinations. The significance of differences between curriculum types within a domain was checked with pairwise exact chi-square tests. Statistical significance was set at the .05 level for all tests. Open-set data were coded into three primary themes and eight sub-themes. Figure 1 details these themes sub-themes and their relationship.

The data collected was intentionally broad in its design to gather information on multiple aspects of the curriculum planning and implementation process. This paper reports on the findings related explicitly to teacher perspectives on curriculum entitlement and the quality of the content and design features of the curriculum (section c of the survey).

## Results

### Participants

The Survey respondents were 46 teachers (95% female, 5% male) of secondary-aged students with severe intellectual disabilities or profound and multiple learning difficulties. Participants were located across multiple Australian states, inclusive of New South Wales (78.3%), Victoria (10.9%), Queensland (6.5%), and Tasmania (4.3%). Table 1 details participant experience in working to support these students.

**Table 1.** Experience and Current Employment Status of Participants.

Current role	Experience (Years working with students with SPMLD)					
	0-1	1-3	4-7	8-10	10+	*Subtotal (role)*
Classroom Teacher: Temporary Contract	1	5	2	0	3	*11*
Classroom Teacher: Permanent	1	3	7	1	5	*17*
Stage/Peer Leader	0	0	0	2	0	*2*
School Executive: Teaching	0	0	3	3	5	*11*
School Executive: Non-Teaching	0	0	0	0	5	*5*
Subtotal (experience)	*2*	*8*	*12*	*6*	*18*	**46**

### Curriculum entitlement

When participants were asked their opinion on how important curriculum inclusion is for all students, the responses were overwhelmingly in favour, with 58.7% of participants stated that curriculum inclusion was ‘extremely' or ‘very' important, while 23.9% responded with ‘somewhat', and 12.8% said this was only ‘slightly' or ‘not at all' important. The remaining, 4.3% of participants chose not to respond. Four primary themes were detailed when participants were asked to comment on why they had made this assessment: inclusive principles, professional support, representation, and relevance.

*Inclusive principles.* In their responses, participants referred to the core principles of inclusion, with reference to normalisation, age-appropriateness and creating a system where students were given the same opportunities as their peers. One participant articulated why this should be a central issue attracting broad-scale support, ‘for that sense of normalisation - these are school kids too!' (t29). Participants saw the development of a curriculum that was inclusive of their students learning needs as a significant step toward educational and societal inclusion.

*Secondary-aged students with SPMLD should be included in the same curriculum framework as this supports inclusion, and these students also need a broad experience of the world or else we are in danger of sectioning them off and making their world effectively narrower than that of others their age (t16)*.

#### Professional support

A quarter of participants who expanded on their initial Likert scale response assumed that a more inclusive curriculum would encourage more professional support and resources to be offered. Participants said a more inclusive curriculum framework would provide greater access to appropriate professional support and that teacher quality and performance would be further sustained by having access to a curriculum that mapped appropriate learning progressions. One participant represented the partnership between relevant curriculum outcomes and the quality of teaching programs, elucidating that without a meaningful curriculum structure, learning progression and lower expectations can emerge, ‘Depending on many factors, [the curriculum is] often unrealistic for students with severe intellectual disabilities or profound and multiple learning difficulties. However, [it is] extremely important learning progressions are occurring rather than just ‘repetition' for the sake of it' (t30).

#### Representation

Participant responses acknowledged that a curriculum genuinely inclusive of all students could lead to all students being seen as valuable contributors to schools and communities, ‘I think it is important for learning progressions to be included for visibility of students with SPMLD within schools' (t8). One participant stated, ‘The learning of students with SPMLD needs to be considered as important as that of their same-aged peers' (t31); indicating that without authentic curriculum inclusion, these students remain less represented in comparison to their same-aged peers.

#### Relevance

A theme repeated throughout responses was the need for relevant learning intentions and outcomes to be developed in the first place. Twelve of the twenty-six participants who contextualised their assessment of importance around curriculum entitlement referred to content appropriateness, rather than structural inclusion, as a primary message in their reasoning. Even in the responses of those who indicated prominent levels of support for the concept, the including relevant outcomes was a critical issue, qualifying their position on the topic by stating, ‘Having appropriate curriculum for our students is highly valuable. Whether or not this is the same format as same-aged peers is somewhat important' (t2). One participant highlighted, ‘It seems like a tokenism to appear we are treating our students equally and [the curriculum] is not relevant for them at all' (t10).

Participants were asked to assess the alignment between their student's IEP goals and current curriculum outcome offerings (state and national). Less than 20% of participants said they could identify links between the IEP goals of their students and the curriculum in most instances. For 30.4% of respondents, they reported that they could identify relevant alignment about half the time, and 34.8% indicated that this is a rare occurrence and rely on a backward mapping process, or stated they could never find a relevant curriculum outcome to the IEP goals developed for students. The remaining 8.7% of participants stated that they specifically use the state curriculum outcomes as their IEP goals.

### Curriculum engagement

Participants were asked how often they engaged with available curriculum documents as part of their planning practice. Although 28.3% of participants reported they do this ‘all of the time' and 30.4% ‘most of the time', over a third (37%) of participants reported they refer to the curriculum only occasionally or less, with 4.3% of participants choosing not to respond. These results are a significant indicator of the reality of the usefulness these teachers feel the curriculum provides them through its clear underutilisation. Some important considerations reinforce the disconnect between teachers, teaching practice and the curriculum when analysing the qualitative contributions with two themes emerging, the absolute requirement to use their state curriculum, and access to a parallel curriculum pathway designed to support students with disabilities.

#### Absolute requirement

Over 60% of responses included references to the obligation to use the curriculum in their reporting and planning requirements or to show the upholding of prescribed accreditation obligations as a primary motivator to engage with the curriculum. The tension discussed earlier between curriculum entitlement and relevance was embedded in many responses. Participants identified two clear ways they managed the conflict between planning and professional obligations, and the need to maintain individual student programs that were meaningful for students. Most participants explained they started with the curriculum to develop programs, adhering to conventional planning expectations, but shared an echoed perspective that ‘the curriculum does not start where these students do’ (t17), and another, ‘I use all the outcomes our state authority requires us to use in our curriculum planning but many of the outcomes and learning activities are not relevant for our students (t9)’.

One-quarter of participants who chose to explain their curriculum engagement practice referred to a process of backwards mapping, focusing first on student needs, planning and implementing their programs, and then post-implementation creating a curriculum relationship in reporting, ‘I often look at what they can do, plan on where they need to go, then look at the curriculum to ensure that I am planning what I need to' (t15), and another stating ‘I find the outcomes to meet what I am doing as a matter of ticking the boxes. The outcomes do not meet the needs of my kids [students]' (t21). Multiple participants referred to the process of curriculum engagement occurring solely because it was a requirement to do so, with 18% of participants who chose to respond to this question explicitly stating they did not engage with the curriculum at all as ‘the outcomes do not meet the needs of my kids' (t21).

#### Parallel curriculum pathways

Participants from all states referred to using a state-endorsed parallel curriculum framework explicitly designed for students with disabilities in their planning practice. Twelve of the 16 participants who referred to their states' parallel offering contextualised its use with the specific need for further adaptation and adjustments for students with severe intellectual disabilities or profound and multiple learning difficulties required, ‘I do A LOT of my own adaptations and adjustments and basically rewrite what is given to me each semester…' (t6), with another acknowledging the lack of relevancy within for-purpose offerings ‘… [The parallel] curriculum doesn't meet the needs of most of the students in my class' (t14).

Teachers in government school settings across Australia are instructed to use their state-identified curriculum documents when planning as the foundational source of the scope and sequence/learning progressions. Only 41.3% of participants indicated they engage with their state curriculum, and 37.5% indicated they use the Australian Curriculum materials (either singularly or most commonly to support other curriculums being used), 7.5% of participants reported using curriculums developed by external providers and 13.8% reported using an internally developed framework. Table 2 shows that participants are using multiple curriculum frameworks and draw on third party unendorsed offerings in their planning.

**Table 2.** Range of Curriculums Accessed by Participants.

Curriculum Type	Participants who use this in their planning practice n(%)
State syllabus/curriculum (State Curriculum)	33(41.2)
The Australian Curriculum (Australian Curriculum)	8(10)
General Capabilities	9(11.2)
Literacy and Numeracy Progressions	10(12.5)
Early Years Learning Framework	3(3.8)
Externally Developed Curriculum (incl. other state syllabi)	6(7.5)
Internally Developed Curriculum	11(13.8)

As well as referring to a diverse range of sources, participants showed significant diversity in the number of curriculum sources they were using at any time. Most participants report using between two and six different curriculum sources in their planning process (52.4%), with 7.1% of these stating that they refer to between four and six different curriculum sources simultaneously.

### Curriculum quality

Participants were asked to rate their current state curriculum's success in meeting the needs of students with severe intellectual disabilities or profound and multiple learning difficulties as shown in Tables 3 and 4. Not one participant assessed their state curriculum as being ‘excellent'. This ‘broad brush' rating of quality was refined when participants were asked to rate the success of their state curriculum in specific areas. These areas included assessing how well the curriculum met the academic needs of their student, personal/well-being and social needs, post-school requirements and living and life skills requirements. Participants were also asked to rate the success of The Australian Curriculum offering and external and internal curriculums used under the same categories.

**Table 3.** Teacher Perceived Success of Curriculums Across five domains of need, mean average (1-5 point Likert scale responses) m(SD).

Domain of Need	State Curriculum	Australian Curriculum	Internally developed	Externally developed	*Overall (Domain)*
Academic need	2.14 (1.04)	2.36 (0.75)	3.22 (0.83)	4.40 (0.55)	*2.9 (0.63)*
Personal/well-being needs	2.16 (1.02)	2.57 (0.65)	3.11 (1.45)	3.80 (1.11)	*2.85 (0.85)*
Social needs	2.00 (1.00)	2.43 (0.76)	3.11 (1.45)	3.40 (0.89)	*2.69 (0.82)*
Post school needs	2.05 (0.97)	2.21 (0.80)	2.44 (1.24)	3.40 (1.14)	*2.42 (0.83)*
Living and life skills	2.14 (0.97)	2.36 (0.93)	3.11 (0.78)	4.00 (1.23)	*2.77 (0.78)*
Overall (Curriculum)	*2.11 (1.00)*	*2.35 (0.78)*	*2.98 (1.15)*	*3.8 (0.98)*	***2.73 (0.78)***

**Table 4.** Teacher Assessed Success of Curriculums as a percentage of respondents that found the curriculum fairly or very successful across 5 domains of need.

Domain of Need	State Curriculum n/43 (%)	Australian Curriculum n/14 (%)	Internally developed n/9 (%)	Externally developed n/5 (%)	Significance*
Academic need	5 (11.6)^a^	0 (0)^a^	4 (44.4)^b^	5 (100)^b^	x^2^(3)=24.0 p < .001
Personal/well-being needs	5 (11.6)^a^	0 (0)^a^	5 (55.6)^b^	4 (80)^b^	x^2^(3)=24.0 p < .001
Social needs	3 (7)^a^	0 (0)^a^	5 (55.6)^b^	3 (60)^b^	x^2^(3)=23.5 p < .001
Post-school needs	3 (7) ^a^	0 (0) ^a^	2 (22.2) ^a^	2 (40) ^a^	x^2^(3)=8.6 p = .03
Living and life skills	2 (4.7)^a^	1 (7.1)^ac^	3 (33.3)^bc^	4 (80)^b^	x^2^(3)=24.5 p < .001

(X)^abc^ -Superscripts to the percentages with the same letter indicate categories that are not significantly different to each other in pairwise comparisons at the .05 significance level. Categories with different letter superscripts are significantly different.

*The significance of the chi-square test was obtained using exact tests as were all the significance tests for pairwise comparisons.

When asked to rank the success of various curriculum documents from 1 to 5, results indicate that participants perceived the most successful type of curriculum across all identified areas are those developed by external providers, with state developed offerings being the least successful. Responses in Table 3 show the mean score given for each curriculum in each domain, giving a nuanced understanding of strengths and relative success within and across the variables.

Table 4 presents the results in each domain as percentages of respondents that indicated a positive bias toward the success of the curriculum using a 5-point Likert scale. Statistically significant chi-square tests were obtained between curricula for each domain. Overall, internally, and externally developed curricula were perceived as more effective than the state or Australian Curriculum offerings in supporting students. The differences between the curriculum groups were most significant for academic needs, living and life skills, and personal/well-being needs, with more minor differences for the other two domains.

The Australian Curriculum attracted no positive rank responses towards its ability to address not only the academic needs of students but also their personal/well-being, social and post-school needs, with all responses only rating it with a 1 or 2 on a 5-point Likert scale. Although slightly more successful than the Australian Curriculum, state curriculums only achieved 11.6% as its highest level of positive rating (Academic and Personal/well-being domains), down to 7% (Social and Post-school domains) and 4.7% (Living and life skills domain). To develop a deeper awareness of why participants made these assessments, they were asked to articulate the strengths and areas of improvement they feel exist within their mandated state curriculum offering.

#### Strengths

When identifying the current strengths within their state curriculums, the most common theme was to refer to the structural provision of the parallel curriculum offerings embedded within the state structures. When analysing participants' responses, it was clear that their structural presence and ability to offer some flexibility, rather than their content, was the primary motivator for inclusion. ‘It does plan for students with disabilities; however, this planning is, from my experience, based on the student with mild intellectual disabilities’ (t22). The proposition of parallel curriculums being a strength within secondary environments was reinforced by references to this kind of option not being available in primary-aged settings in some states, ‘The Life Skills [NSW] areas for students in secondary school are broad and can generally be applied to students, but these students need access to this kind of program earlier on' (t17).

#### Areas for improvement

Participants identified diverse areas for improvement and how the curriculum could be changed to best include students with severe intellectual disabilities or profound and multiple learning difficulties. The most common suggestion was the inclusion of outcomes relevant to theses students' capacities and needs. There was advocacy for ‘[the inclusion of] *MEANINGFUL* [emphasis authors] age-appropriate curriculum with appropriate, relevant, and realistic outcomes that can be used across [specialist settings] and schools that cater for students with SPMLD’ (t6). The term meaningfulness was explored and qualified by many. Some advocated for the inclusion of outcomes responsive to the needs of non-verbal students, transition to work and post-school lives, and students with pre-foundational core literacy and numeracy skills while maintaining age-appropriateness for high school students. Structural changes were also sighted, with some participants indicating a move away from key learning area (KLA) planning and towards domain planning, focusing on communication, well-being, and independence to develop a more accessible and meaningful structure.

*There is a significant dilemma on whether we focus on teaching our students with severe intellectual disabilities the life skills content of effective communication, social skills, and self-care such as dressing independently and toileting, to name a few, or whether we continue to just focus on academic content (t15)*.

Multiple participants referenced the need for more sensitive assessment and tracking tools, with one participant stating, ‘Some of our students are not even able to achieve one outcome in Stage 5 and 6 mathematics Life Skills syllabus' (t31) and another, ‘Life Skills etc. has great detail but for [students with] severe [disabilities] no realistic ideas [are] put forth to attend to the curriculum as well as really understand that sometimes moving an eyebrow is progress' (t29). Increasing the structural support available to teachers is also reflected in suggestions for specific exemplars of scope and sequences, working proformas and illustrations of practice that articulate best practices, and other forms of administrative support and guidance specific to the context of specialist settings. Although there is deep thinking and reflection about workable solutions, there are also elevated levels of pessimism about the current offering and trust in the validity of future improvements, with multiple participants stating that that the way to resolve the issue is to ‘start again' (t25), and ‘For the writers to actually enter a special school and realise the students they are writing about. They clearly have no idea' (t27).

## Discussion

This research provides insight into the curriculum planning and design practices of teachers of students with severe intellectual disabilities or profound and multiple learning difficulties and their professional assessment of the curriculum itself. Results show a broadly negative assessment of the current curriculum's capacity to be inclusive for students and that their teachers need help finding any relevant or meaningful aspects to its design or implementation.

In investigating the research question of; *How are the currently endorsed curriculums and supporting documents perceived, adjusted and/or operationalised by teachers who support students aged 12-19 with severe intellectual disabilities or profound and multiple learning difficulties who attend specialist settings?,* we have found that from the experience of teachers, Australia has a curriculum offering that is not only unsupportive of teachers but one of minimal relevance for the educational growth and prosperity of students with severe intellectual disabilities or profound and multiple learning difficulties. Systems design the curriculum to be the backbone of educational experiences for all. It is intended to be utilised in teachers' day-to-day and long-term planning and sets nationally consistent standards for educational opportunity and growth (NESA, 2022b). In its current form, the curriculum in Australia is unsuitable when analysing its capacity to support teachers in the educational, social, and cultural inclusion of students with severe intellectual disabilities or profound and multiple learning difficulties. The tensions that arise in the national standardisation of curriculum and curriculum entitlement continue to be played out in other OECD countries, with the work of Butler (2021) examining the current and historical challenges within the United Kingdom (UK), which are ahead of Australia on the nationalised curriculum journey, with the UK’s National Curriculum being introduced in 1989, 25 years prior to Australia’s adoption of a similar structural approach beginning in 2014. In the UK, these tensions and debates are still very much active and still without resolution or agreement on ways forward (Butler, 2021). It is suggested that the experiences of both Sweden and Norway are also examined, with both countries having a more fully realised inclusive educational curriculum framework and a longer-established policy and practice dedication to mainstream inclusion of all students while also sharing similar social and economic conditions to Australia (Fasting, 2010; Flem & Keller, 2010; Ostlund, 2015). Both Sweden and Norway maintain some forms of segregated schooling, and the work of Goransson et al. (2022) and Magnusson et al. (2019) reveal there are distinct commonalities shared between Sweden and findings herein regarding the navigation of systems and teacher perspectives of the current structural and curriculum offering (Flem & Keller, 2010). By looking towards these examples and shared challenges, even within a more supported and longer established inclusive policy framework, Australia may be able to gain the capacity to articulate and respond, and therefore address these as we move forward. If Australia is to improve its current system, we can use the path set by others to gain insight on the road ahead, and learn from their experiences.

The inclusion of relevant educational pathways for students with severe intellectual disabilities or profound and multiple learning difficulties within the curriculum is a crucial step toward recognising the value of these students in our schools and our community. For teachers, a curriculum structure that enables them to see themselves and their work as a part of the teaching community reduces feelings of disengagement or aloneness and is vital to teacher sustainability and engagement (Rendoth et al., 2023). Advocating for a curriculum structure that promotes rather than ignores the needs of students with severe intellectual disabilities or profound and multiple learning difficulties will improve the system for all and enable teachers to implement more effective individualised yet consistent and relevant educational experiences for their students.

### Trends of concern

In the data, there was a cluster of concerning trends regarding the navigation of the reporting and planning requirements placed on teachers. These include using the backward mapping process, IEP and curriculum goal correlations, and non-age-appropriate tools for curriculum planning.

An interesting finding was the presence of backward mapping in a considerable number of teachers curriculum planning practices. Although allowing for the specific individualisation and reclaiming the narrative of student led teaching in a standardised system, there are some significant drawbacks to this practice (McDonald, 1992). If not done with sensitivity, backward mapping may be a process that ignores the curriculum content throughout the teaching planning and implementation phases and falsely claims connectivity at reporting time, which can undermine the tenant of curriculum entitlement as a central structural level of inclusion. This is further emphasised when paired with the acknowledgement of these teachers working over many curriculum frameworks. A backwards mapping approach to planning, although meeting the central needs of individualisation, can promote an environment for teachers to feel even more unsupported in how to navigate next steps or provide a pathway for growth (Cho & Trent, 2005). Backward mapping is also a process that could also increase work hours, planning complexities, and compound the atmosphere of responsibility and isolation between the teacher and the educational system (Strnadova et al., 2022; Timberlake, 2016; McDonald, 1992). From a systems perspective, the practice of backward mapping allows the inadequacy of the current offering to be unrealised as reporting mechanisms reflect false effectiveness and an ability for teachers to meet curriculum entitlement requirements.

The crucial importance of IEPs and their role in tailoring educational programs for these students, the group of teachers who specifically use curriculum outcomes to develop IEPs is of deep concern (8.6%). In the search to find any connection to the curriculum to maintain reporting and planning obligations, teachers may use widely unviable general curriculum outcomes. Using general curriculum outcomes explicitly in the IEP process undermines its role and value, and reflects a progression toward further standardisation, disabling the IEP's capacity to inject meaning and purpose into the student's individual educational journey.

Non-engagement with the curriculum raises potential interactions with the quality, consistency, and reliability of a student's individual curriculum, as well as adding time to workload responsibilities if teachers are operating solely off individualised plans that they are self-developing and resourcing. Of major concern is that over 40% of participants report rarely engaging with their state curriculum at all, which leaves the question, if not the curriculum, what are they using to support their practice and planning? (Franklin, 2017). This is a research area for the future, but a fact that reinforces and reveals the widespread perception of the lack of structural support for this group of teachers.

In the study, we learned that 16.3% of survey participants indicated drawing from ACARA's Early Years Learning Framework (EYLF) and Literacy and Numeracy Progressions (LNPs) (see Table 2), which are designed to be used in the pre-formal and early years of learning. All participants of the study worked to support secondary-aged students (12-19 yrs. old), and although developmentally relevant, neither the EYLF nor the LNPs are age-appropriate or designed in any way to relate to the experiences of a teenager or young adult. Age-appropriateness is a cornerstone of inclusion and must be upheld if schooling is enriching across academic, emotional, social, and cultural domains of learning and experience but can pose a nuanced challenge for student-centred planning (Forster, 2010; Strnadova et al., 2021). With the importance of post-school life increased in the later years of schooling, it is essential to question how these pre-formal documents relate to the needs of students or support teachers in working towards an independent life beyond the classroom. The impact of age as a defining feature of curriculum is important to consider for teachers when navigating infantilisation practices, socio-cultural age expectations and connectivity to identity and life worlds of students, depriving them of recognition of lived experience and a supported transition into adulthood, while also enabling interests and developmental capacities as a primary consideration (Mietola & Vehmas, 2019).

The use of pre-formal documents, IEP goal standardisation creep, and backwards mapping contradict the overall system design framework but are inevitable if teachers cannot see any relevance or benefit in meeting and maintaining curriculum alignment. The quality assessment of the various curriculum offerings gives insight into why teachers are engaging in these behaviours. Of particular concern is the overall quality assessment given to state offerings, with an assessment of 2.11 out of a possible 5. This is a concerning assessment of state authorities' current curriculums for teachers to support students with severe intellectual disabilities or profound and multiple learning difficulties, particularly when looking at the capacity to prepare students for post-school and whole-of-life engagement in their community.

### Learning from others

How we enable our standardised system to evolve through investigating and learning about these already existing curriculum frameworks is something that needs to be seriously considered and progressed. Respondents continually referred to the need for curriculums to be made more meaningful and include more appropriate academic and personal development outcomes. By examining the quality ratings given to the internally and externally developed curriculum frameworks, findings reveal that teachers feel there are curriculum options available that offer higher quality levels of support and appropriateness compared to Australia’s current provisions.

## Limitations

The participant base was drawn from teachers working in publicly funded specialist settings. Broadening the workplace location would offer a larger participant base and provide the opportunity to compare experiences across the settings to assess whether all teachers of students with severe intellectual disabilities or profound and multiple learning difficulties similarly feel the experiences reported herein or if it is specific to those working in Specialist schooling environments. Another limitation was that the survey was only open for a four-month period and was released halfway through the second COVID-19 lockdown in Australia, which could have limited the enthusiasm for engagement. There were limitations related to recruitment, the use of peak organisational bodies as primary distribution partners. Although offering extensive support to the research team and ensuring research relevance, researchers were restricted in recruitment reach. Using peak organisations as distribution partners meant that the research team could only contact participants indirectly, reducing the potential participant pool by focusing on the membership of these bodies as a primary recruitment pathway.

## Conclusion

There is clear majority support for full curriculum entitlement; and a prominent level of willingness to offer guiding insights and workable solutions in beginning this process. A judgement can be made that this is a cohort of teachers who care deeply for their students and their profession, despite their negative assessment of their current curriculum and system context. When further research is conducted, the research team strongly recommends that teachers’ voices play a significant role in the research process and urge the crucial importance of the intertwined nature of context (teacher, school, community) and curriculum (Jackson et al., 2008).

It is clear from the results herein that there are already curriculums more closely aligned to the strengths and needs of students with severe intellectual disabilities or profound and multiple learning difficulties being utilised by teachers compared to what is currently being offered by state authorities or through the Australian Curriculum, be they developed internally at a school level, or externally developed by either private enterprise or other educational systems/departments internationally. The participant base is resoundingly in support of an inclusive curriculum framework. It is important that the extensive body of research that has been conducted in countries outside of Australia is examined, with sensitivity to the development of an inclusive curriculum framework structure (Jones, 2017; Rendoth et al., 2022; Thompson et al., 2018).

This study has revealed that teachers of secondary-aged students with severe intellectual disabilities or profound and multiple learning difficulties operate within an environment of curriculum exclusion, complexity, and irrationally ineffective systemic support that needs to be acknowledged and remediated. This research reveals that Australia's curriculum offering cannot cultivate nor sustain educational inclusion for all students or support the teaching professionals that work within. With a curriculum structure so inaccessible and inappropriate for teachers and students alike, the issue of exclusion is systemic. These issues extend across the philosophical, structural, academic and implementation domains of curriculum design and combine to restrict the capacities of teachers to design and implement appropriate or meaningful curriculums for secondary-aged students with severe intellectual disabilities or profound and multiple learning difficulties.

Curriculum reform is attainable with the professional and practice insights from teachers, the structural and content inclusions gained from other curricula, and a dedicated focus from researchers. This complex but compulsory reform process needs urgent attention underlined by the joint attention of researchers, policymakers, and teaching professionals alike.

## Supplemental Material

Supplemental Material - Curriculum effectiveness for secondary-aged students with severe intellectual disabilities or profound and multiple learning difficulties in Australia: Teacher perspectives Supplemental Material for Curriculum effectiveness for secondary-aged students with severe intellectual disabilities or profound and multiple learning difficulties in Australia: Teacher perspectives by Tess Rendoth, Jill Duncan and Judith Foggett, Kim Colyvas in Journal of Intellectual Disabilities
