# Supplementary material for: Curriculum effectiveness for secondary-aged students with severe intellectual disabilities or profound and multiple learning difficulties in Australia: Teacher perspectives
Source: J Intellect Disabil. 2024 Jan 24;29(4):852–68. doi: 10.1177/17446295241228729 (PMC12672946; doi:10.1177/17446295241228729)
Supplement: Supplemental Material - Curriculum effectiveness for secondary-aged students with severe intellectual disabilities or profound and multiple learning difficulties in Australia: Teacher perspectives [file sj-pdf-1-jld-10.1177_17446295241228729.pdf]

Appendix A

Survey Questions

Provided below is a list of the specific questions asked of participants related to the results discussed within this article.

Please note that these questions comprised a fraction of a larger survey that sought information responding to all three research questions, including more data not yet reported, related to the focus question for this article, *how are the currently endorsed curriculums and supporting documents perceived, adjusted and/or operationalised by teachers?*

For the purposes of readability, questions have been re-arranged into like-for-like groupings, rather than in their original truncated structures. No language has been changed or amended.

For a full list of the questions included in the survey, please contact the first author.

**Question**	**Quantitative**	**Qualitative**
Pre-survey screening questions		
I am currently employed as a teacher (in any capacity, permanent, temporary, casual) in an Australian government school that provides education to students with disabilities exclusively	Yes/No	
I have curriculum planning responsibilities for secondary-aged students (12-19 years) with severe or profound learning difficulties or multiple disabilities	Yes/No	
Demographics		
I identify as…	Tick box	
Which Australian State or territory do you work in	Tick box	
What best describes your geographic location	Tick Box	
What best describes your main role at school	Tick Box	
How long have you been<br>…teaching in a specific special education environment<br>…working with students with SPMLD<br>…working in your current school	Multiple choice	
How many students in your class have a diagnosis of SPMLD?	Multiple choice	
Questions reported on within this Article		
What curriculum documents do you refer to when planning for your students?<br>…your state education authority<br>…your state curriculums alternative for students with disabilities (if offered).<br>…The Australian Curriculum (including the general capabilities/literacy and numeracy progressions and EYLF)<br>…Externally developed curriculum provider<br>…Internally developed curriculum framework guides<br><br>NOTE: "No" responses removed any reference to this curriculum type throughout the rest of the survey. "Yes" responses meant that the survey section related to each of these curriculum types was served to the participant.	Yes/No	

From now on these different types of curriculums will be referent to as (Curriculum Type) for the purposes of readability.		
How often do you refer to<br>…the curriculum of your state authority<br>… The Australian Curriculum<br>to facilitate your planning form students with SPMLD	5-point Likert scale	Comment box
Rate the curriculum and planning advice provided by (Curriculum Type).	5-point Likert Scale plus n/a option	
How important is it that the learning progressions of secondary-aged students with SPMLD are included in the same curriculum framework as their same-aged peers?	5-point Likert scale	Comment box
How successfully do you think that (Curriculum Type)<br>is addressing the following needs of your students…?<br>…Academic<br>…Personal and well-being<br>…Social<br>…Post-school<br>…Living/Life Skills	5-Point Likert scale	
Rate your level of confidence in…<br>…Locating the documents and materials of (Curriculum type)<br>…navigating the advice provided by (Curriculum type)<br>… Understanding your obligations of (Curriculum type)<br>… Following the planning and structural advice (Curriculum type)<br>…Applying to suit the needs of your students<br>…Applying it in the classroom	5-point Likert scale	
How closely aligned are the outcomes of (Curriculum Type) and the personal learning goals you set for your students?	5-point Likert scale	Comment box
What are the strengths of (Curriculum Type)?		Open response
What are the opportunities for improvement of (Curriculum Type)?		Open response
